# Pollen Carbon-Based Rare-Earth Composite Material for Highly Efficient Photocatalytic Hydrogen Production from Ethanol–Water Mixtures

**DOI:** 10.1021/acsomega.2c03949

**Published:** 2022-08-19

**Authors:** Xia Jiang, Yan-Xin Chen, Jing-Wen Zhou, Shi-Wei Lin, Can-Zhong Lu

**Affiliations:** † CAS Key Laboratory of Design and Assembly of Functional Nanostructures, and Fujian Provincial Key Laboratory of Nanomaterials, Fujian Institute of Research on the Structure of Matter, Chinese Academy of Sciences, Fuzhou 350002, P. R. China; ‡ Xiamen Key Laboratory of Rare Earth Photoelectric Functional Materials, Xiamen Institute of Rare-earth Materials, Haixi Institutes, Chinese Academy of Sciences, Xiamen 361021, P. R. China; § College of Chemistry and Materials Science, Fujian Normal University, Fuzhou, Fujian 350007, P. R. China; ∥ School of Chemistry and Chemical Engineering, Jiangxi University of Science and Technology, Ganzhou 341000, P. R. China

## Abstract

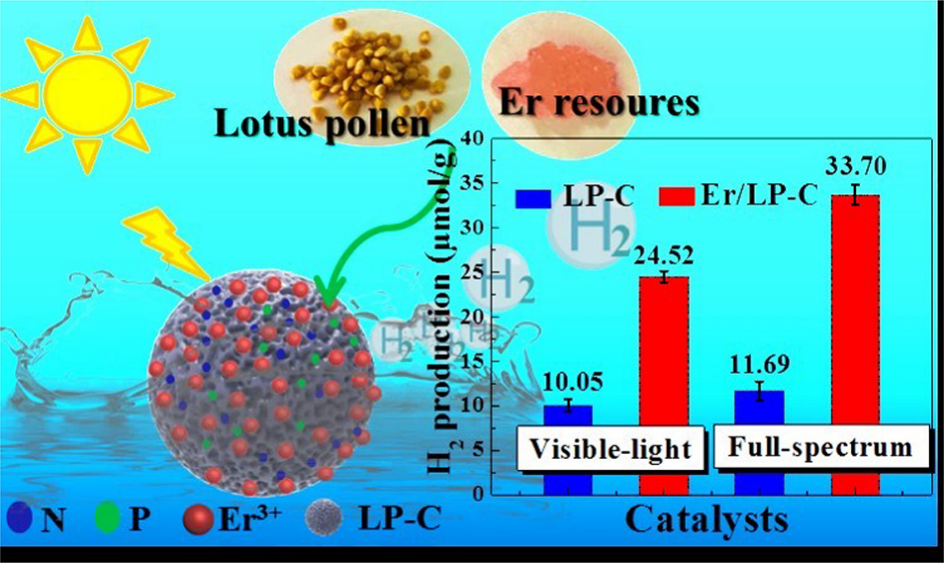

The unique electronic structure of rare-earth elements makes their modified semiconductor photocatalysts show great advantages in solar energy conversion. Herein, the pollen-like N, P self-doped biochar-based rare-earth composite catalyst (Er/LP-C) has been successfully synthesized, which combines the advantages of biochar and Er and is used for the first time in the field of photocatalytic hydrogen production from ethanol–water mixtures. Experimental results confirmed that the performance of photocatalytic hydrogen production under the full spectrum is up to 33.70 μmol/g in 6 h; this is due to the introduction of Er, which improves the carrier concentration, separation and transfer efficiency, and the driving force for the reduction reaction.

## Introduction

The realization of photocatalytic water splitting to produce hydrogen is an important issue for the conversion of solar energy into chemical energy.^1^ Although many types of catalysts (such as inorganic semiconductors, organic carbon-based photocatalysts, semiconductor coordination compounds, etc.) have been studied,^2^ their catalytic performance is far from practical applications, and the synthesis process of catalysts with better catalytic performance is often complicated and costly, which is not conducive to large-scale production. Therefore, it is necessary to search for a high-efficiency catalyst that has a simple, economical preparation process and can be mass-produced.

Carbon materials have an important position in photocatalysis, because they possess characteristics of large specific surface area, good electrical conductivity, and high chemical and thermal stability and can be used as adsorption sites and activation centers of reactants, electron-receiving and transfer channels, cocatalysts, photosensitizers, supports, and so on.^3^ Biochar, as a kind of carbon material, not only has the virtues of the above-mentioned carbon materials but also has an obvious advantage in the economy for it is derived from natural biomass.^4^ At the same time, biochar can inherit the morphology and porous structure of natural biomass and realize the self-doping of elements (P, N, etc.) to improve the use of light, enhance the mass transfer, and raise the dispersion of the loaded metal.^5,6^ Excitingly, biochar can be directly used to realize the composite with other substances for improving the electron transfer.^7−9^ However, the current research on biochar-based photocatalysts is mainly focused on the degradation of organic pollutants,^4,10,11^ and there is less research in photocatalytic hydrogen production from water.^6,8,9,12−15^ Moreover, it is difficult to achieve a high catalytic effect using a simple carbon material.

Rare-earth elements are rich in reserves, own a unique electronic structure, possess optical and magnetic properties, and have many applications in scientific research and industry.^16^ The upconversion characteristics of rare-earth elements can broaden the light absorption region, and their unique electronic structure is conducive to the rapid transfer of electrons, making their modified inorganic semiconductor photocatalysts show great advantages in solar energy conversion.^17,18^ However, the current use of rare-earth elements is mainly focused on doping into photocatalysts, and there is little research on the supported rare-earth materials.^19−21^ In addition, under our knowledge, the research on the photocatalytic hydrogen production of catalysts loaded with rare-earth materials has not been reported.

In this work, lotus pollen with a wrinkled surface that could improve light utilization^22,23^ was chosen as a template together with erbium nitrate to prepare a pollen-like N, P self-doped biochar-based rare-earth composite material (Er/LP-C) through the process of simple mixing and two-step calcination. The reason for choosing Er is that it has advantages such as high chemical stability, upconversion luminescence efficiency in the visible and ultraviolet regions, etc., among various rare-earth elements.^24^ Meanwhile, the effects of pollen carbon on the loading of Er and the loaded rare-earth substances on the photocatalytic hydrogen production from ethanol–water mixtures were investigated.

## Experimental Section

### Materials

Erbium nitrate (Er(NO_3_)_3_·5H_2_O) from Aladdin Reagent Co., Ltd. (China), was used as an Er precursor. Lotus pollen was supplied by Wutaishan Industry Co., Ltd. (China). Anhydrous ethanol was purchased from Sinopharm Chemical Reagent Co., Ltd. (China).

### Preparation of the LP-C and Er/LP-C Catalysts

**Figure 1:**
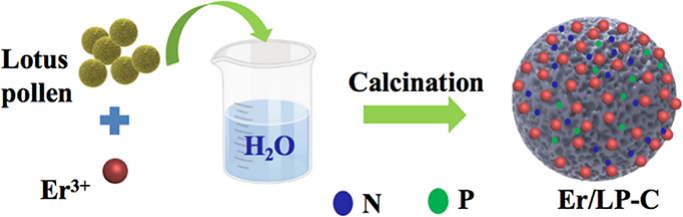
Schematic diagram of the synthetic method.

The preparation process of lotus pollen carbon (LP-C) and erbium-modified lotus pollen carbon (Er/LP-C) catalysts is shown in Figure 1. First, the purchased lotus pollen (20 g) was pretreated by washing with ethanol (200 mL) under sonication for 10 min, then dried at 60 °C for 12 h, and marked as LP-Et. Second, 2 g of LP-Et materials was further placed in a muffle furnace for annealing under air at 300 °C for 6 h, with a ramp rate of 5 °C min^–1^. The samples were then transferred into a tube furnace for carbonization at 600 °C with a heating rate of 10 °C min^–1^ for 3 h under argon. After washing and centrifuging with ultrapure water for five times, LP-C catalysts were then dried at 60 °C overnight for use.

The preparation process of Er/LP-C materials was obtained as follows: First, 2 g of LP-Et samples was slowly mixed with a certain volume of Er (NO_3_)_3_·5H_2_O aqueous solution (100 mL). After continuous stirring for 24 h and following centrifugal washing (5000 r/min, 5 min) with ultrapure water and ethanol successively, the samples were then dried at 60 °C for 24 h. After that, similar annealing and post-treatment methods were used for obtaining LP-C (annealing under air at 300 °C for 6 h with a 5 °C min^–1^ ramp rate; follow-up annealing under argon at 600 °C for 3 h with a heating rate of 10 °C min^–1^ for carbonization). The final black powder was marked as Er/LP-C. The obtained Er/LP-C materials with 5, 10, 15, 20, and 30% erbium loadings were related to the Er(NO_3_)_3_·5H_2_O aqueous solution concentrations of 2.8, 5.6, 8.4, 11.2, and 16.8 g/L, respectively.

### Characterization

The morphology and composition of the samples were taken by a field emission scanning electron microscope equipped with an energy spectrum (Apreo S LoVac, CZ, Thermo Fisher, Waltham, MA, USA) and a high-resolution transmission electron microscope (TECNAI F30, Philips-FEI, NB, Eindhoven, Netherlands). The surface functional groups of the samples were characterized by the Fourier transform infrared (FTIR) spectra, which were measured by an infrared spectrophotometer (Nicolet iS 50, Thermo Fisher, Waltham, MA, USA). The X-ray diffraction (XRD) system (Miniflex 600, Akishima, Rigaku, Tokyo, Japan) was used to characterize the crystal phase and structure of the synthesized materials. Additionally, the test was performed in the 2θ range of 10–90° with a scan rate of 5°/min with Cu Kα (λ = 0.15406 nm) scan. The surface chemical species, states, and content of the samples were analyzed by X-ray photoelectron spectroscopy (XPS, Scientific K-Alpha, Thermo Fisher, Waltham, MA, USA) using monochromated Al Kα (1486.8 eV) as an X-ray source, and all binding energies were calibrated by the C 1s peak at 284.8 eV. Raman spectroscopy (DXR 2Xi, Thermo Fisher, Waltham, MA, USA) was used to study the structure of as-prepared materials. The elemental content of samples was determined by inductively coupled plasma optical emission spectrometry (ICP-OES, Agilent 5110, Agilent Technologies Inc., CA, USA) and elemental analysis (EA, Elementar Vari EL Cube, Langenselbold, GER). The PL spectra were acquired using a fluorescence spectroscopy test system (PL, FLS980, Edinburgh Instruments Ltd., Livingston, UK).

### Electrochemical (EC) and Photoelectrochemical (PEC) Performance

All the electrochemical and photoelectrochemical tests were carried out in a standard quartz-made three-electrode cell, in which the Pt foil and Ag/AgCl/Cl electrode (saturated KCl) were used as the counter electrode and reference electrode, respectively. The work electrode was obtained by spin-coating the Nafion alcohol suspension (5 wt %) containing the photocatalysts (10 mg) on the fixed area (1 × 1 cm^2^) of the FTO glass. The 0.1 M Na_2_SO_4_ aqueous solution (pH = 6.5) was used as the supporting electrolyte, which was deaerated by bubbling high-purity Ar for 15 min before EC/PEC measurements.

Linear sweep voltammetry (LSV) and chronoamperometry (*I*–*t*) measurements were carried out in a photoelectrochemical test system (PEC2000, Beijing Perfectlight Technology Co., Ltd., Beijing, China), which is connected with a conventional electrochemical workstation (CHI 760E, Shanghai Chenhua, Shanghai, China). A 300 W xenon lamp equipped with a filter (AM 1.5G) and a power density of 100 mW/cm^2^ (PLS-FX300HU, Beijing Perfectlight Technology Co., Ltd., Beijing, China) was used as an illumination source. Photocurrent ON/OFF cycles were measured using the PEC2000 photoelectrochemical test system coupled with a mechanical chopper. Typically, LSV measurements were performed in the potential range of 0.0 to 1.0 V (vs Ag/AgCl) with a scan rate of 5 mV s^–1^. Chronoamperometry measurements were conducted at 1.0 V (vs Ag/AgCl) under intermittent or constant illumination.

Moreover, the monochromatic incident photon-to-electron conversion efficiency (IPCE), Mott–Schottky, and electrochemical impedance spectroscopy (EIS) measurements were carried out in an IPCE1000 photo-electrochemical test system (Beijing Perfectlight Technology Co., Ltd., Beijing, China) equipped with an electrochemical station (CS 350H, Wuhan Corrtest Instrument Corp., Ltd., Wuhan, China).

### Photocatalytic (PC) Performances

The performance of photocatalytic hydrolysis hydrogen production was characterized by an MCP-WS1000 Photochemical workstation (Beijing Perfectlight Technology Co., Ltd., Beijing, China) equipped with a 50 mL quartz-made reactor under artificial solar irradiation. Specifically, 20 mg of as-prepared photocatalysts was ultrasonically dispersed (30 min) into a solution consisting only of 15 mL of ethanol and 15 mL of water (pH = 7), which were deaerated by vacuuming for 10 min before H_2_ evolution measurements. The full spectrum source is simulated sunlight consisting of nine LED lamps (365, 385, 420, 450, 485, 535, 595, and 630 nm LEDs and one white light LED, which is 420–750 nm), and the power of the total light irradiation was around 100 mW/cm^2^. The visible light source consists of nine white light LED lamps (420–750 nm) with a total visible light irradiation power of 100 mW/cm^2^. The temperature of the reaction was controlled by circulating condensed water at 5 °C throughout the reaction process using a water-cooling system. The produced hydrogen was calculated by the external standard method using the peak area obtained by a PLD-CGA1000 composite gas analyzer (Beijing Perfectlight Technology Co., Ltd., Beijing, China).

## Results and Discussion

**Figure 2:**
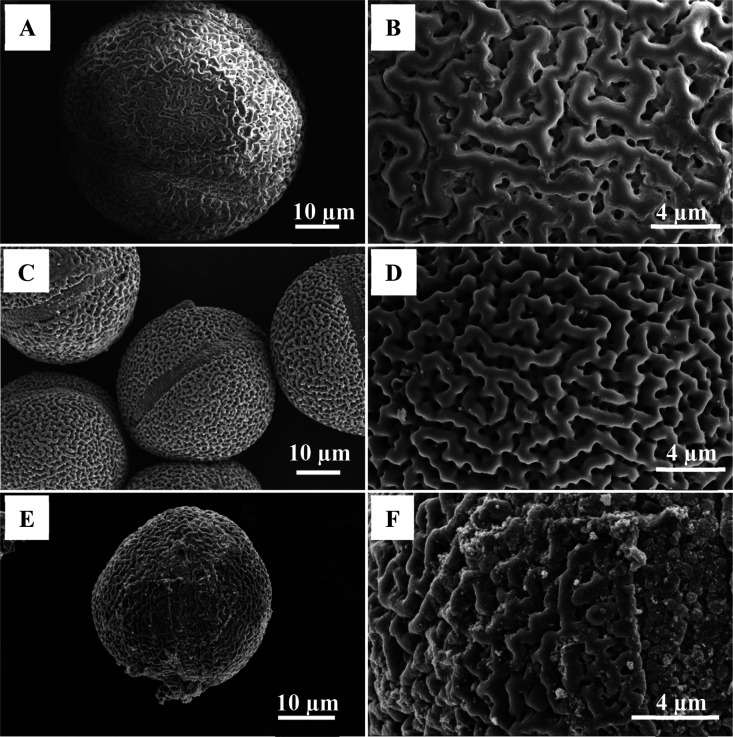
SEM images of LP-Et (A, B), LP-C (C, D), and 20% Er/LP-C (E, F).

The whole preparation process of Er/LP-C is just mixing pollen with Er^3+^ in an aqueous solution for a certain time, which is then cleaned and burned (Figure 1). Among them, the roasting temperature of pollen is selected according to the results of TG and catalyst evaluation (Figures S1 and S2). SEM results show that the raw pollen is spheroidal with a wrinkled surface (Figure 2A,B). Compared with the raw pollen, LP-C shows the similar morphology, but the diameter is smaller than that of the original pollen, which may be caused by the shrinkage of the inner core and the outer shell after roasting,^25^ and there is still an obvious gully on the surface (Figure 2C,D). Similarly, Er/LP-C obtained by adding Er can still maintain the spherical shape and gully surface (Figure 2E,F). These irregular surfaces can improve the light utilization of the catalyst. In addition, combining the results of calculation of the specific surface area of pollen-C obtained using the BET method (almost 0 m^2^/g) and the results of SEM, it can be seen that the obtained materials basically possess a macroporous structure.

**Figure 3:**
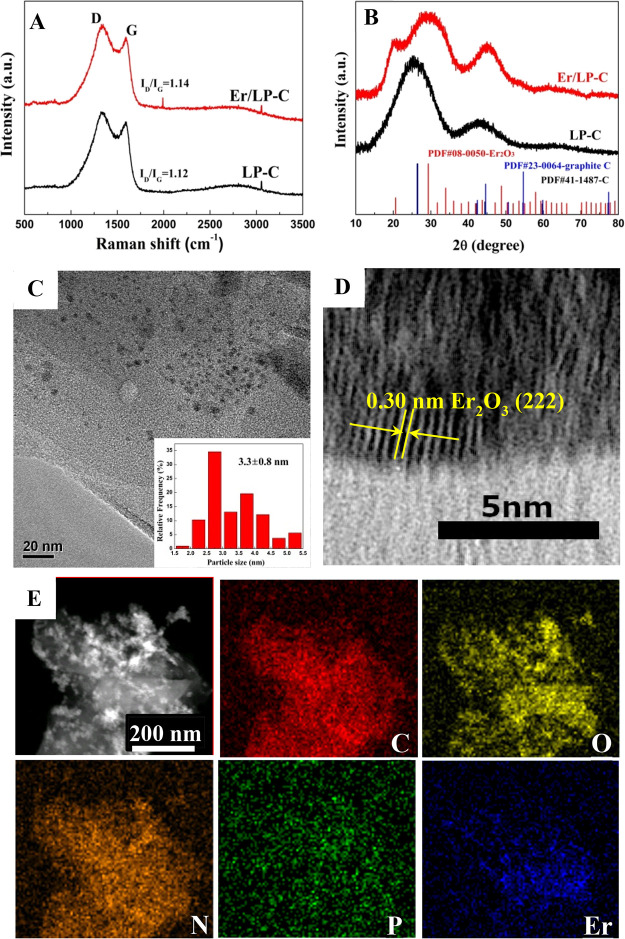
Raman spectra of samples (A); XRD patterns of samples (B); TEM image of Er/LP-C and particle size distribution (C); high-resolution TEM image of Er/LP-C (D); HAADF image of Er/LP-C and elemental mapping images of C, O, N, P, and Er (E). The added mass ratio of Er relative to LP is 20%.

Next, the structure and composition of samples were characterized in detail. It can be seen from the Raman spectra (Figure 3A) that both samples present characteristic peaks of the D band (about 1340 cm^–1^) and G band (about 1590 cm^–1^),^26^ and the intensity ratios of D band to G band (*I*_D_/*I*_G_) are almost identical, which indicates that the introduction of Er does not change the degree of graphitization of biochar, indirectly indicating that Er is not incorporated into biochar but may be loaded on biochar in the form of an element or oxide. The XRD pattern shows that LP-C has obvious wide diffraction peaks at around 26° and 43°, indicating that the obtained pollen carbon has a certain C structure (PDF#41-1487), which is consistent with the Raman results. When Er was introduced, the wide diffraction peaks at around 26° and 45° were consistent with the graphitized structure (PDF#23-0064), while the peaks at 21° and 29° were consistent with cubic Er_2_O_3_ (PDF#08-0050), demonstrating that the use of this method can generate Er_2_O_3_/biochar materials. TEM was used to test the structure and composition of the obtained composite materials. It is found that the material composited with biochar shows a spherical particle shape of about 3.3 nm (Figure 3C). The HR-TEM image further confirms the existence of Er_2_O_3_, in which the lattice fringes with a distance of about 0.30 nm correspond to the (222) crystal planes of Er_2_O_3_ (Figure 3D). Moreover, the high-angle circular dark-field transmission microscope equipped with EDX mapping shows that the sample contains not only C and Er but also N, O, and P elements, and the distribution is relatively uniform (Figure 3E). Referring to the preliminary test results of EDS (Figure S3), the contents of several elements were analyzed by ICP-OES and EA. The test results showed that the main element contents of C, N, P, and Er in 20% Er/LP-C are 35.59, 5.79, 5.41, and 26.84%, respectively (Table S1).

**Figure 4:**
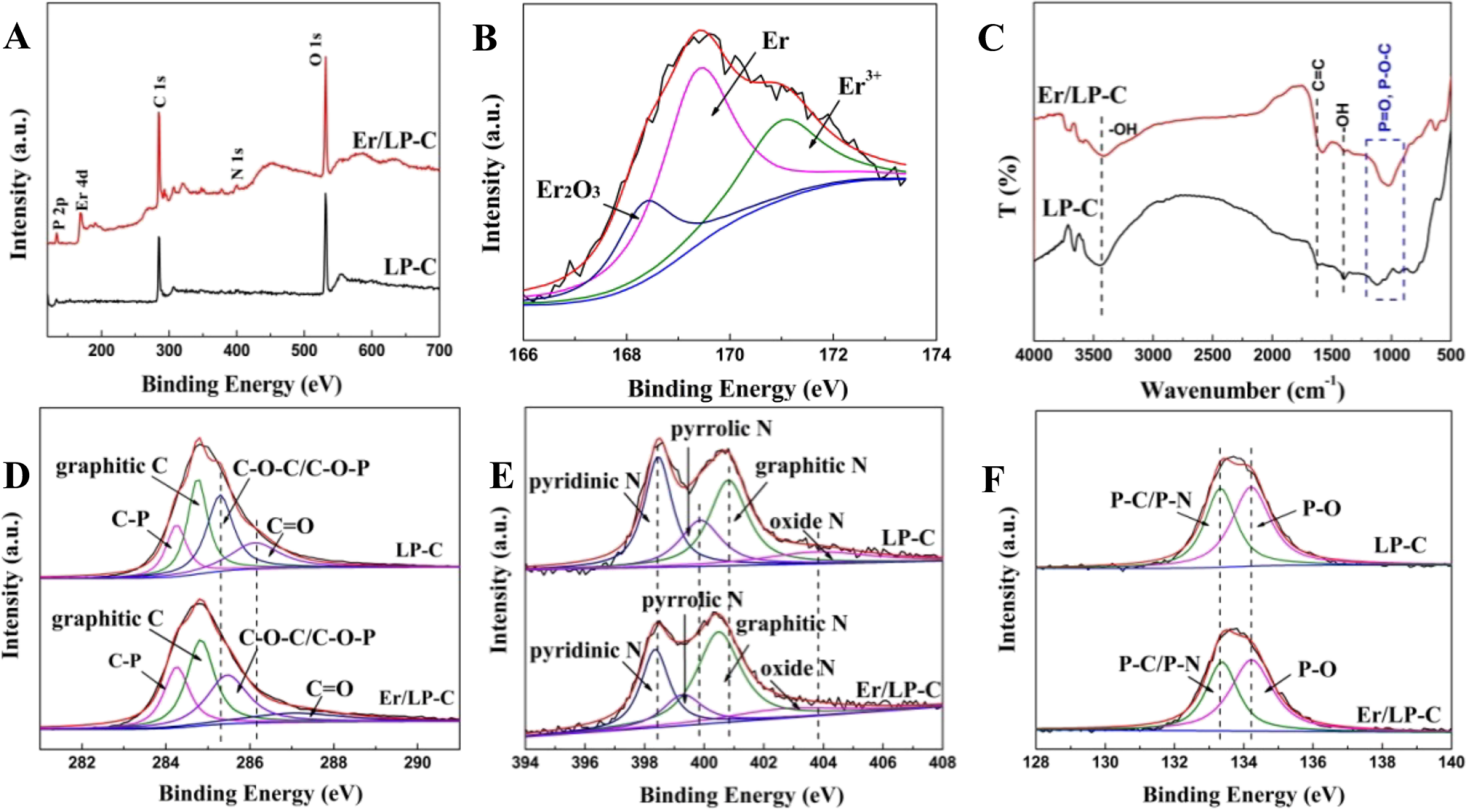
XPS full survey spectra of samples (A); high-resolution spectra of Er 4d (B); FTIR spectra of samples (C); high-resolution spectra of C 1s (D), N 1s (E), and P 2p (F). The added mass ratio of Er relative to LP is 20%.

XPS results further confirmed the existence of N, P, and Er elements (Figure 4A). Also, as shown in Figure 4B, the high-resolution spectrum of Er can be divided into three peaks, which are assigned to Er_2_O_3_ (168.3 eV),^19^ Er (169.4 eV),^19^ and Er^3+^ (171.0 eV),^20^ indicating that Er is combined with biochar, which may be in the form of an elemental substance (Er) and oxide (Er_2_O_3_). The abundant electronic structure of Er may be related to its interaction with biochar. FTIR spectroscopy and XPS were used to study the interaction mode between Er and biochar in detail. FTIR spectroscopy shows that there are many functional groups such as C=C on biochar (Figure 4C). When Er is combined with biochar, the stretching vibration of C=C (1623 cm^–1^)^27^ of LP-C shifts to 1581 cm^–1^, and the stretching modes of P=O and P–O–C (900–1200 cm^–1^)^28^ also have certain changes, indicating that Er may be loaded by interacting with the C- or P-containing functional groups on the surface of biochar. However, it is difficult to discern from FTIR spectroscopy whether the load of Er is related to N-containing substances or not. The high-resolution C 1s XPS spectra reveal that the binding energy of C–P (284.1 eV)^26^ has not changed after the introduction of Er, but the binding energies of C–O–C/C–O–P (285.3 eV) and C=O (286.1 eV)^29^ are shifted to 285.5 and 287.1 eV, respectively (Figure 4D), which indicates that Er may have a certain effect on C-containing substances. For the XPS N 1s spectra, the binding energies of pyridine N (398.4 eV), pyrrolic N (399.9 eV), graphitic N (400.8 eV), and oxide N (403.8 eV)^30^ are shifted to 398.3, 399.3, 400.5, and 402.7 eV, respectively, after loading Er (Figure 4E), which means that Er has a certain effect on N-containing species. Also, in the XPS P 2p spectra, P-related binding energies (P–O, 134.2 eV; P–C/P–N, 133.3 eV)^29,31^ remain basically unchanged after loading Er (Figure 4F), indicating that the introduction of Er has little to do with the P species. The above results indicate that Er interacts with C- and N-containing groups on the surface of biochar to affect the electronic structure of the material.

**Figure 5:**
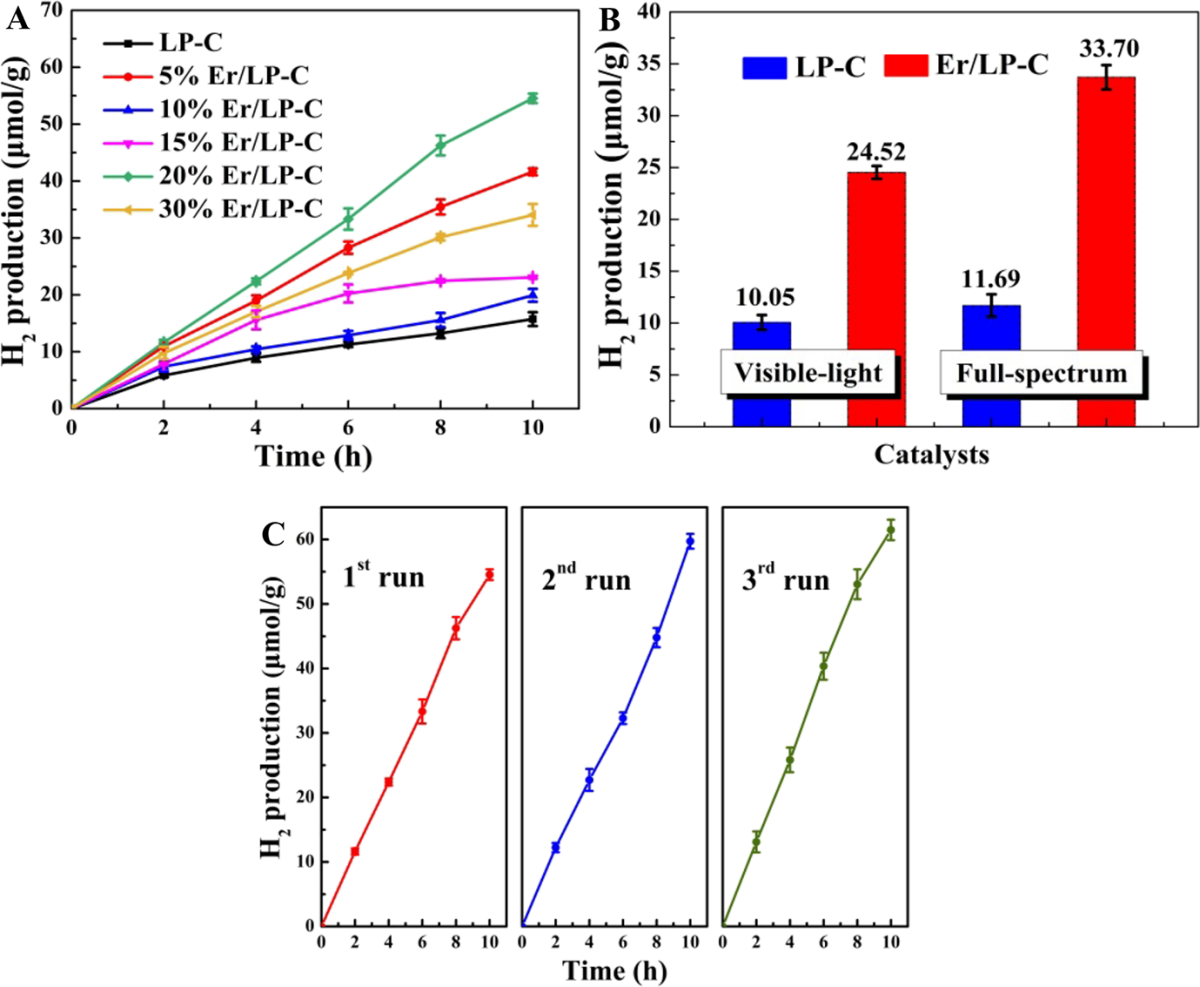
Photocatalytic hydrogen performance of samples under simulated sunlight (A). Photocatalytic hydrogen performance of samples under visible light and simulated sunlight in 6 h (B). The added mass ratio of Er relative to LP is 20%. Stability tests over the 20% Er/LP-C catalyst under simulated sunlight (C).

The obtained composite material (Er/LP-C) was used for photocatalytic hydrogen generation. It can be seen from Figure 5 that the introduction of Er can improve the effect of photocatalytic hydrogen production, and the hydrogen production performance is optimal when the added mass ratio of Er relative to LP is 20% (Figure 5A), which may be due to the suitable dispersion and loading of Er species. In addition, compared with LP-C (11.69 and 10.05 μmol/g), the introduction of the rare-earth element Er can improve the hydrogen production performance to 33.70 μmol/g in 6 h under simulated sunlight and 24.52 μmol/g in 6 h under visible light (Figure 5B). Moreover, compared with the addition of ethanol, the catalytic performance of both LP-C and Er/LP-C in the absence of ethanol was lower (Figure S4). Furthermore, after three cycle tests under the same conditions, Er/LP-C still maintains good hydrogen production performance, indicating that it possesses better stability (Figure 5C). To explore the relationship between the introduction of Er and the improvement of catalytic performance, further photoelectric performance tests were carried out.

**Figure 6:**
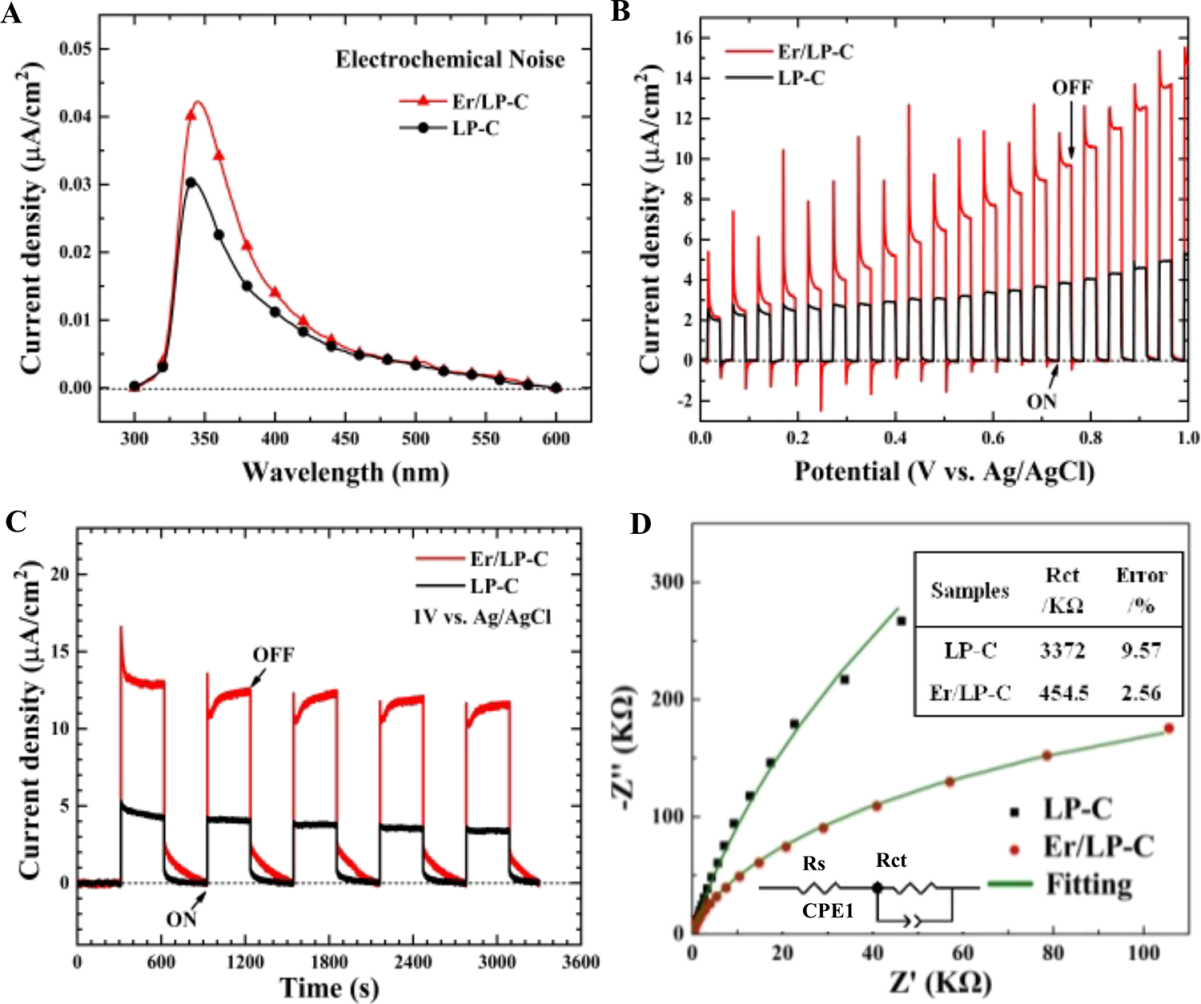
Photocurrent density of samples under monochromatic light (A); linear sweep voltammetry (LSV) curves of samples (B); transient photocurrent responses of samples (C); electrochemical impedance spectra of samples (D). The inset shows the fitted equivalent circuit and its impedance parameters. The added mass ratio of Er relative to LP is 20%.

**Figure 7:**
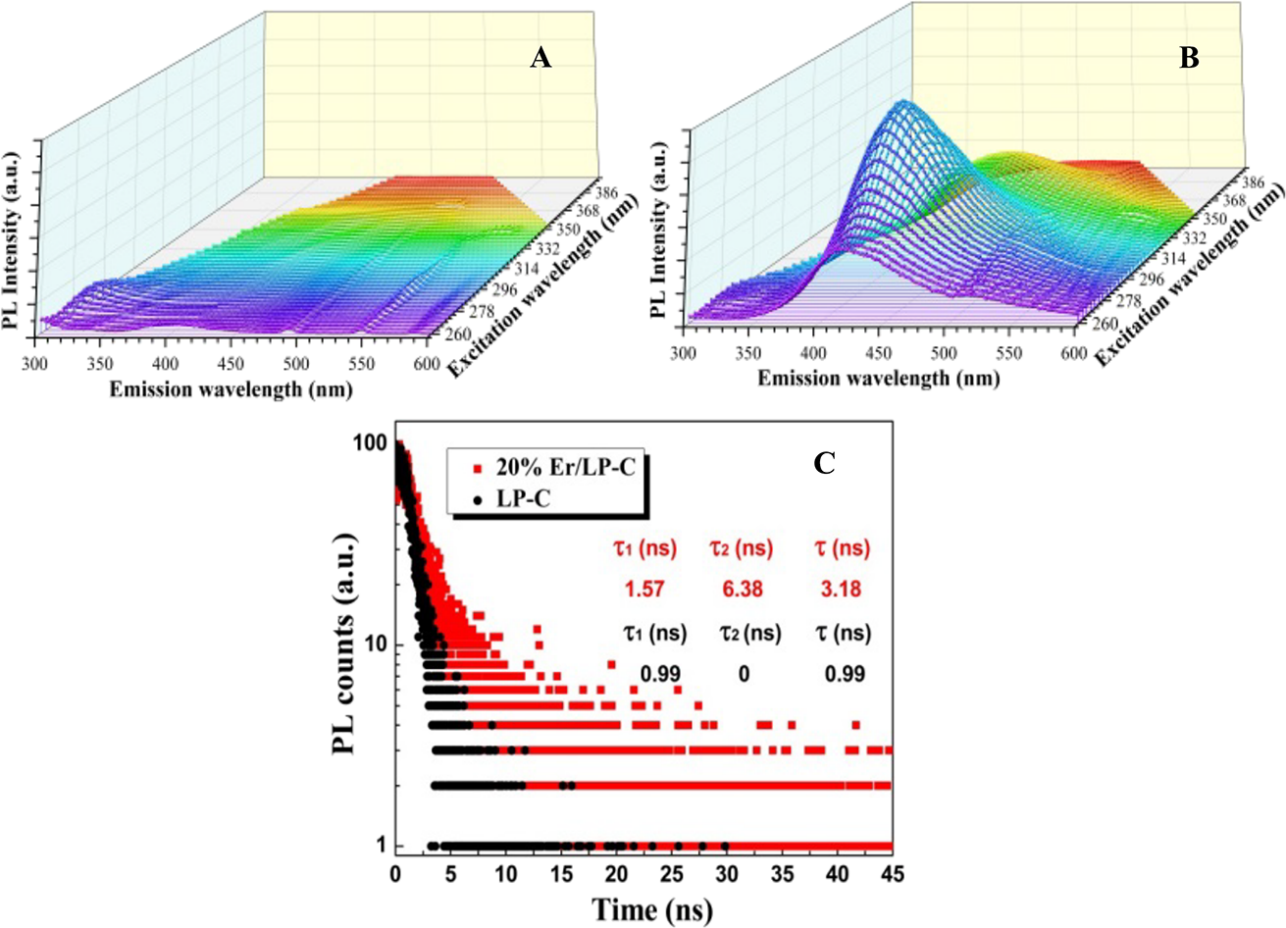
Photoluminescence (PL) emission spectra of LP-C (A) and 20% Er/LP-C (B); time-resolved PL spectra of samples (C).

The photocurrent density of catalysts in the monochromatic light test results shows that the light absorption range of the two catalysts is around 300–600 nm, which will be more conducive to the use of sunlight, and the photocurrent generated by the introduction of Er is significantly higher than that of biochar (Figure 6A). This may be the reason why the introduction of Er improves the catalytic performance under both visible light and simulated sunlight. Linear sweep voltammetry (LSV) and transient photocurrent responses were used to detect the photogenerated current response. As shown in Figure 6B,C, Er/LP-C obtains a much higher photocurrent than LP-C, indicating that the introduction of Er can improve the separation efficiency of photogenerated carriers. At the same time, the samples have good stability. Furthermore, we investigated the photocurrent transfer speed of samples by EIS. The radius of the semicircle in the Nyquist plots is proportional to the charge transfer resistance,^32^ so it can be seen from Figure 6D that the charge transfer resistance of Er/LP-C is smaller, and it is found by fitting results that compared with LP-C (3372 KΩ), Er/LP-C (454.5 KΩ) possesses less resistance to charge transfer, which endows Er/LP-C with excellent carrier separation and charge transfer ability during the photocatalysis. The charge behavior was further analyzed by PL; it can be seen from Figure 7 that after adding Er, the fluorescence intensity is significantly enhanced, which indicates that the Er-modified sample produces more photogenerated electron–hole pairs under illumination.^33^ Also, under the same excitation wavelength, the fluorescence intensity under the same emission wavelength is different, which indirectly confirms that Er species has a greater contribution to the emitted fluorescence. At the same time, referring to the absorption map of the sample for monochromatic light (Figure 6A), the fluorescence emitted by the Er-modified sample can be reabsorbed and utilized. In addition, the fluorescence lifetime of 20% Er/LP-C is longer than that of LP-C, indicating that the introduction of Er can improve the separation ability of carriers (Figure 7C). In summary, although it can be seen from the PL diagram that Er/LP-C has a large number of carrier recombination, the light emitted by it can be reused. Combined with the results of catalytic performance and other electrochemical characterization results, it shows that the introduction of Er species can generate more available photogenerated carriers and help improve the hydrogen production performance.

**Figure 8:**
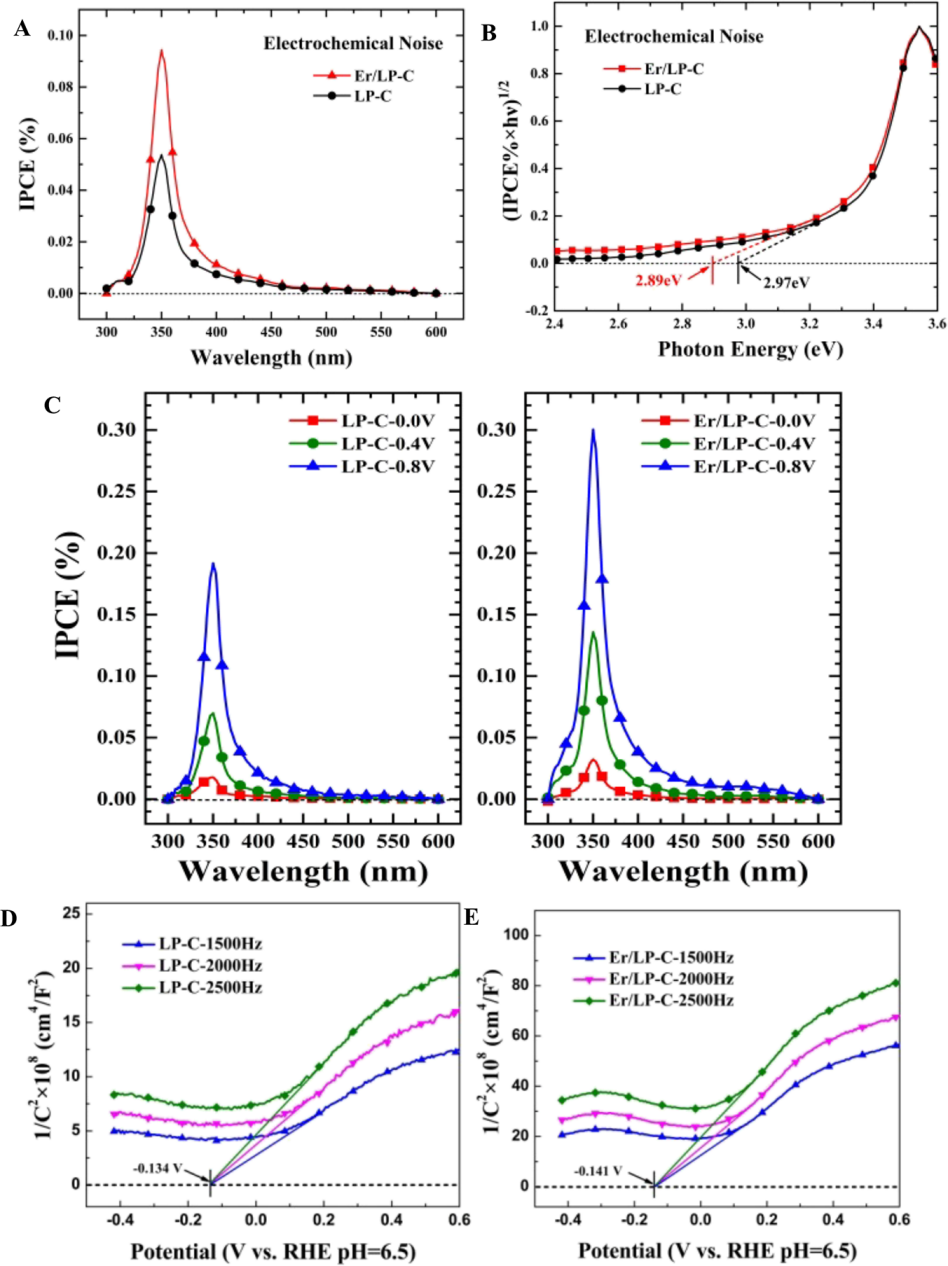
IPCE (%) spectra of samples under monochromatic light (A); band gap determination extracted from IPCE (%) (B); IPCE (%) spectra of samples under monochromatic light and certain bias voltage (C); Mott–Schottky plots of LP-C (D) and Er/LP-C (E). The added mass ratio of Er relative to LP is 20%.

The IPCE (%) values of LP-C and Er/LP-C are approximately 0.05 and 0.09 at about 350 nm as shown in Figure 8A. Moreover, although a relatively accurate band gap can be obtained by using UV–vis DRS (Figure S5), most of the light absorbed under this band gap cannot generate electrons due to the black color of the sample. The IPCE spectra are used to obtain the available band gaps (*E*_g_) of catalysts based on the assumption that the number of absorbed photons (that is, the absorption efficiency) is proportional to the photocurrent density;^34^ as shown in Figure 8B, both LP-C and Er/LP-C have narrow band gaps (2.97 and 2.89 eV, respectively), which correspond to the fact that the samples can generate photocurrents in the visible light region. It is worth mentioning that after adding bias voltage, the obtained photocurrent density (Figure S6) and IPCE (Figure 8C) under monochromatic light irradiation have been greatly improved, which indicates that Er/LP-C will exhibit much greater hydrogen production performance when the bias voltage is present. The position of the semiconductor conduction band similar to the flat band potential^35^ is shown in the Mott–Schottky curve (Figure 8D,E). It can be seen that the Er/LP-C sample exhibits a more negative flat band potential (−0.141 V vs. −0.134 V) compared with the LP-C sample, indicating that the electron has the strongest reducing ability, which is more conducive to the photocatalytic hydrogen production reaction. In addition, the obtained materials are n-type semiconductors according to the result of Mott–Schottky curves (Figure 8D).

To sum up, it is worth noting that although the photocatalytic hydrogen production rate of the materials obtained in this paper is lower than that reported in many literature (Table S2), the catalysts in this paper still show a certain hydrogen production effect without noble metals on the condition that the preparation method is simple and economical, and the obtained material can acquire photogenerated carriers at broad wavelengths (300–600 nm). At the same time, although the specific reaction mechanism needs to be further studied, it has been confirmed that Er species has a certain contribution to the performance of photocatalytic hydrolysis for hydrogen production, so it has a certain research value.

## Conclusions

In this paper, a biochar-based rare-earth composite material (Er/LP-C) was successfully prepared using pollen that has a porous structure and is rich in hetero elements (N and P) as raw materials. Its performance of photocatalytic hydrogen production under the full spectrum is up to 33.70 μmol/g in 6 h. This is due to the fact that the introduction of Er can change the energy band distribution of the catalyst, thereby increasing the carrier concentration and separation and transfer efficiency and improving the driving force of the reduction reaction. This study not only provides new directions for the utilization of natural biomass but also provides new ideas for the design and development of rare-earth photocatalysts.

## Supporting Information Available

Additional data regarding the TG spectrum of LP-Et under Ar; photocatalytic hydrogen performance of LP-C under different calcination temperatures; EDS of LP-C; table for the elemental content of LP-C and 20% Er/LP-C determined by ICP-OES and EA; photocatalytic hydrogen performance of samples under simulated sunlight without ethanol; UV–vis DRS of samples; photocurrent density of samples under monochromatic light and certain bias voltage; table for reported biochar-based photocatalysts for water splitting (PDF)
