# Supplementary material for: Pollen Carbon-Based Rare-Earth Composite Material for Highly Efficient Photocatalytic Hydrogen Production from Ethanol–Water Mixtures
Source: ACS Omega. 2022 Aug 19;7(34):30495–503. doi: 10.1021/acsomega.2c03949 (PMC9434610; doi:10.1021/acsomega.2c03949)
Supplement: Supplementary file 1 — ao2c03949_si_001.pdf [file ao2c03949_si_001.pdf]

Supporting Information

Pollen carbon-based rare-earth composite material for highly efficient photocatalytic hydrogen production from ethanol-water mixtures

Xia Jiang [a,b], Yan-Xin Chen [a,b*], Jing-Wen Zhou [a,b,c], Shi-Wei Lin [a,b,d] and Can-Zhong Lu [a,b*]

[a](CAS Key Laboratory of Design and Assembly of Functional Nanostructures, and Fujian Provincial Key Laboratory of Nanomaterials, Fujian Institute of Research on the Structure of Matter, Chinese Academy of Sciences, Fuzhou 350002, China)

[b](Xiamen Key Laboratory of Rare Earth Photoelectric Functional Materials, Xiamen Institute of Rare-earth Materials, Haixi Institutes, Chinese Academy of Sciences, Xiamen 361021, China)

[c](College of Chemistry and Materials Science, Fujian Normal University, Fuzhou, Fujian 350007, P. R. China)

[d](School of Chemistry and Chemical Engineering，Jiangxi University of Science and Technology, Ganzhou 341000, P. R. China)

***Corresponding authors**

Email to: yanxinchen@fjirsm.ac.cn (Yan-Xin Chen) and czlu@fjirsm.ac.cn (Can-Zhong Lu)

According to the TG curve (Figure S1), when the roasting temperature reaches 500 ^{o}C, the quality of the sample basically does not change, therefore, in the pre-test, the roasting temperature of 500 ^{o}C, 600 ^{o}C, 700 ^{o}C and 800 ^{o}C were selected to obtain pollen carbon. The photocatalytic hydrogen production performances at several calcination temperatures were investigated under same conditions, and it was found that the pollen carbon obtained by calcining at 600 ^{o}C had the best performance (Figure S2), so this temperature was selected as the calcination temperature for obtaining pollen carbon.

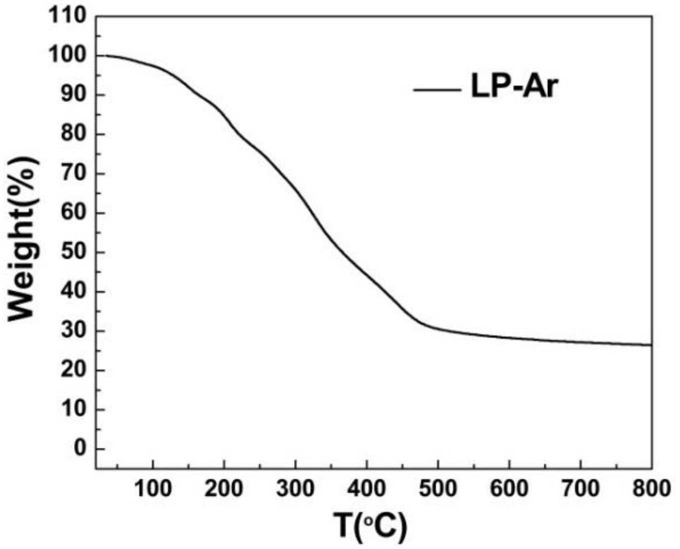

Figure S1. TG spectrum of LP-Et under Ar.

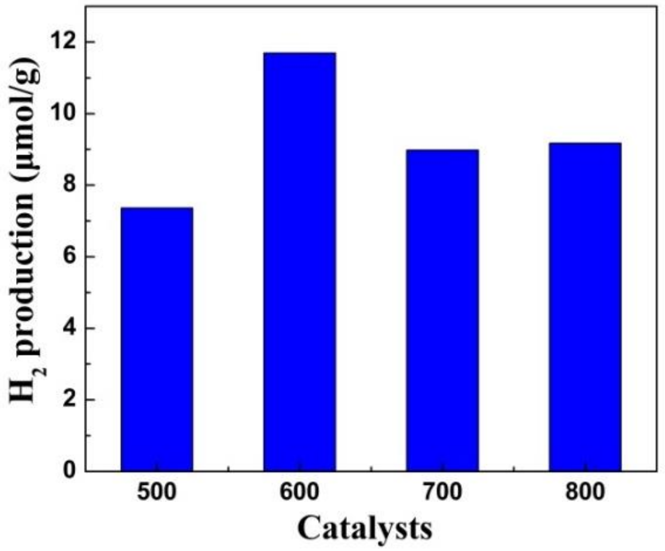

Figure S2. Photocatalytic hydrogen performance of LP-C under different calcination temperature (condition: 20 mg catalyst, simulate sunlight in 6 h).

The element content of the obtained pollen carbon has been detected by EDS (Figure S3), the elements present are mainly C, N, O and P, and a small amount of K and Mg.

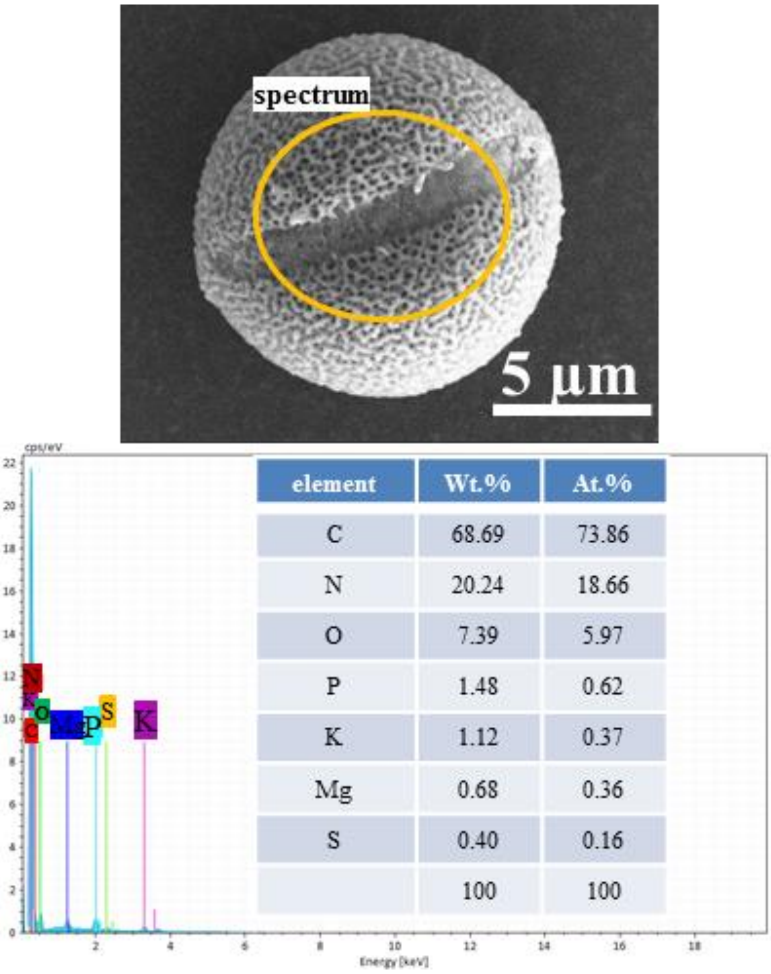

Figure S3. The EDS of LP-C

Table S1. Elemental content of LP-C and 20%Er/LP-C determined by ICP-OES and EA

Samples	C[2]	H[2]	N[2]	O[2]	P[1]	S[1]	S[2]	K[1]	Mg[1]	Er[1]
LP-C	60.51%	2.037%	7.50%	14.94%	0.12%	0.01%	0.37%	0.35%	0.09%	0%
20%Er/LP-C	35.59%	1.61%	5.79%	15.23%	5.41%	0.02%	0.32%	0.05%	0.06%	26.84%

[1] determined by ICP-OES

[2] determined by EA

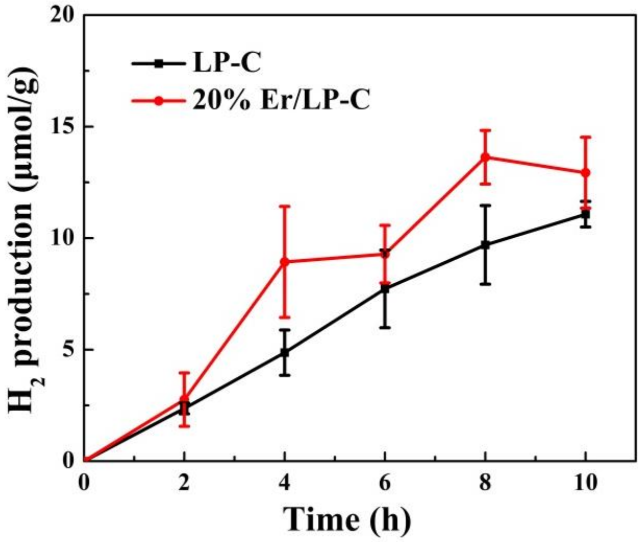

Figure S4. Photocatalytic hydrogen performance of samples under simulate sunlight without ethanol

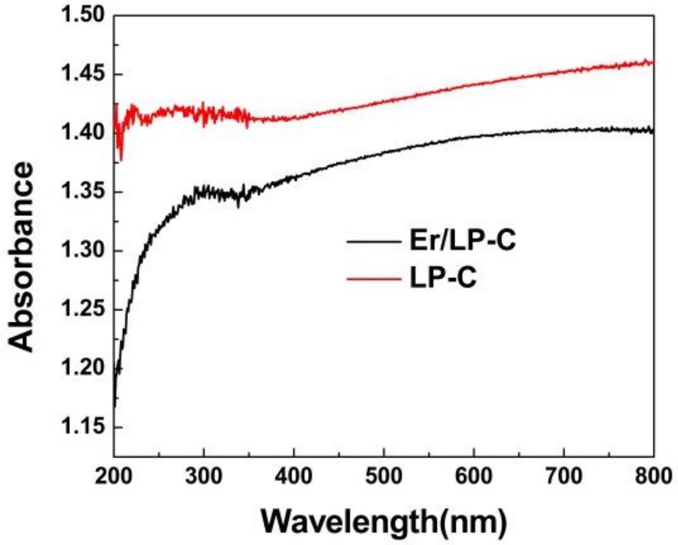

Figure S5. UV-vis DRS of samples

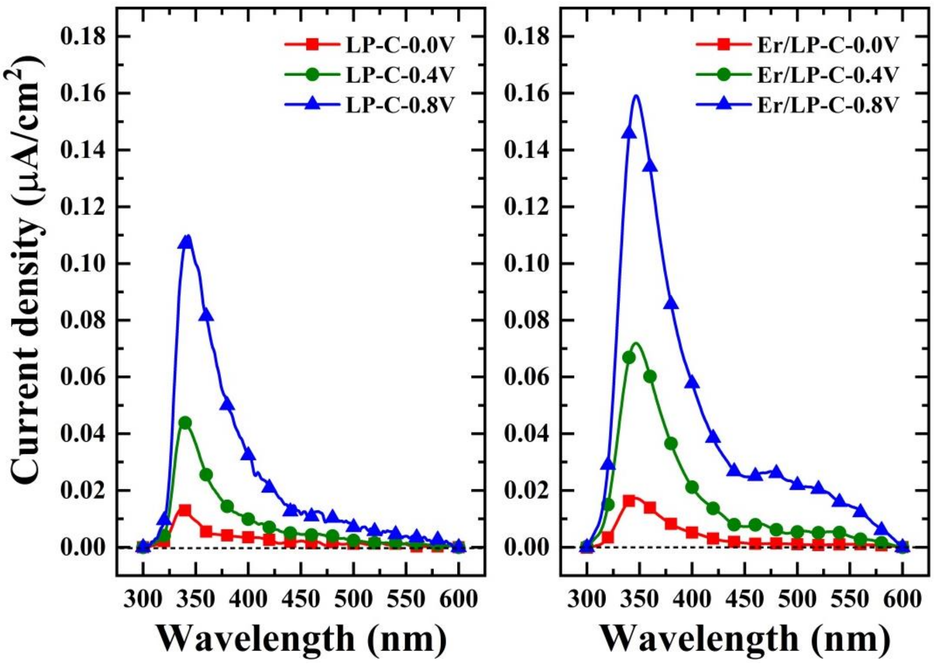

Figure S6. Photocurrents density of samples under the monochromatic light and certain bias voltage

We summary the properties and reaction parameters of the biochar-based photocatalysts used in the field of photocatalytic hydrolysis for hydrogen production. As shown in Table S2, the light intensity and sacrificial agent used in the current reaction are quite different, which will inevitably lead to different catalytic performance, so it is difficult to compare catalysts in different literatures.[7] Although the photocatalytic hydrogen production rate of the materials obtained in this paper is lower than that reported in many literatures, the catalysts in this paper still show a certain hydrogen production effect without noble metal and on the condition of the preparation method is simple and economical, and the obtained material can acquire photogenerated carriers at broad wavelengths (300-600 nm). At the same time, although the specific reaction mechanism needs to be further studied, it has been confirmed that Er species has a certain contribution to the performance of photocatalytic hydrolysis for hydrogen production, so it has certain research value.

Table S2. Reported biochar-based photocatalysts for water splitting

Samples	Dosage/ mg	Cocatalys	Light source	Light power/ mW·cm^{-2}	Reactant solution	H_2 envolution/ μmol g^{-1} h^{-1}	Photoelectric range/nm	Ref.
Pt/CdS/CSC	102.4	Pt/CdS	300 W Xe lamp/ λ>420 nm	Null	20 vol% lactic acid/100 mL	16401.37	Null	(1)
Pt/CSC	2.4	Pt				0		
CeO_2 QDs/carbon	100	CeO_2QDs	300 W Xe lamp	50	10 vol % methanol/100 mL	582	Null	(2)
Nano-CeO_2/biocarbon		Nano-CeO_2				≈100		
1.5CCN650	10	Pt	Xe lamp/ λ>420 nm	100	10 mL triethanolamin/100 mL	1889	Null	(3)
SS700	40	Null	300 Xe lamp/ λ>420 nm	Null	10 vol % of triethylamine/40 mL	1281.67		
SWP700	40	Null	300 Xe lamp/ λ>420 nm	Null	10 vol % of triethylamine/40 mL	848	Null	(4)
RH700	40	Null	300 Xe lamp/ λ>420 nm	Null	10 vol % of triethylamine/40 mL	1038.33		
BZF-5	20	$ZnFe_2O_4$	300 W Xe lamp>420 nm	Null	5 vol% TEOA, 1.5 mM EY/20 mL	1219	Null	(5)

Table S2. Continued

Samples	Dosage/ mg	Cocatalys	Light source	Light power/ mW·cm^{-2}	Reactant solution	H_2 envolution/ μmol g^{-1} h^{-1}	Photoelectric range/nm	Ref.
CWB-CuCs	100	Cu^{2+} -doped carbon Spheres	Null	Null	20 mL TEOA /100 mL	1900	Null	(6)
LP-C	20	Null	9 LED lamps Simulate sunlight	100	15 mL ethanol/30 mL	1.95	300-600 nm	This work
Er/LP-C	20	Er species	9 LED lamps Simulate sunlight	100	15 mL ethanol/30 mL	5.62	300-600nm	

(1) Zha, D. W.; Li, L. F.; Pan, Y. X.; J. B. He. Coconut shell carbon nanosheets facilitating electron transfer for highly efficient visible-light-driven photocatalytic hydrogen production from water. *Int. J. Hydrogen Energ.* **2016**, *41*, 17370-17379.

(2) Qian, J. C.; Chen, Z. G.; Sun, H.; Chen, F.; Xu, X.; Wu, Z. Y.; Li, P.; Ge, W. J. Enhanced photocatalytic H_2 production on three-dimensional porous CeO_2/carbon nanostructure. *ACS Sustainable Chem. Eng.* **2018**, *6*, 9691-9698.

(3) Zhang, L. H.; Jin, Z. Y.; Huang, S. L.; Huang, X. Y.; Xu, B. H.; Hu, L.; Cui, H. Z.; Ruan, S. C.; Zeng, Y. J. Bio-inspired carbon doped graphitic carbon nitride with booming photocatalytic hydrogen evolution. *Appl. Catal. B: Environ.* **2019**, *246*, 61-71.

(4) Norouzi, O.; Kheradmand, A.; Jiang, Y. J.; Maria, F. D.; Masek, O. Superior activity of metal oxide biochar composite in hydrogen evolution under artificial solar irradiation: A promising alternative to conventional metal-based photocatalysts. *Int. J. Hydrogen Energ.* **2019**, *44*, 28698-28708.

(5) Chen, D.; Wang, X. N.; Zhang, X. Q.; Yang, Y.; Xu, Y.; Qian, G. G. Facile fabrication of mesoporous biochar/$ZnFe_2O_4$ composite with enhanced visible-light photocatalytic hydrogen evolution. *Int. J. Hydrogen Energ.* **2019**, *44*, 19967-19977.

(6) Zhou, Y. L.; Sun, M.; Yu, T.; Wang, J. 3D g-C_3N_4/WO_3/biochar/Cu^{2+}-doped carbon spheres composites: Synthesis and visible-light-driven photocatalytic hydrogen production. *Mater. Today Commun.* **2022**, *30*, 103084.

(7) Zheng, W.; Li, C.; Domen, K. Recent developments in heterogeneous photocatalysts for solar-driven overall water splitting. *Chem. Soc. Rev.* **2019**, *48*, 2109-2125.
